# Crystal structure of di-μ-chlorido-bis­[di­chlorido(l-histidinium-κ*O*)cadmium(II)]

**DOI:** 10.1107/S205698901900690X

**Published:** 2019-05-17

**Authors:** Nacira Mohamedi, Slim Elleuch, Sihem Boufas, Messaoud Legouira, Faiçal Djazi

**Affiliations:** a Laboratoire de Géni-méncanique et Matériaux, Faculté de Technologie, Université de Skikda, 21000, Algeria; b Laboratoire de Physique Appliquée, Faculté des Sciences de Sfax, Université de Sfax, BP 1171, 3000 Sfax, Tunisia; c Laboratoire de Recherche sur la Physico-chimie des Surfaces et Interfaces, Université de Skikda, 21000, Algeria

**Keywords:** X-ray diffraction, hybrid materials, dinuclear cadmium compounds, hydrogen bonds, electronic properties, crystal structure

## Abstract

In the title compound, [Cd_2_(C_6_H_9_N_3_O_2_)_2_Cl_6_], the coordination polyhedra around the Cd^II^ cations are distorted trigonal bipyramids. Two of the chloride ions (one axial and one equatorial) are bridging to the other metal atom, leading to a Cd⋯Cd separation of 3.9162 (4) Å. The O atom of the l-histidinium cation lies in an axial site. In the crystal, numerous N—H⋯Cl, N—H⋯O, C—H⋯O and C—H⋯Cl hydrogen bonds link the mol­ecules into a three-dimensional network. Theoretical calculations and spectroscopic data are available as supporting information.

## Abstract 2

In the bimetallic title compound, [Cd_2_(C_6_N_3_O_2_H_9_)_2_Cl_6_], both cadmium atoms adopt a distorted CdCl_4_O trigonal–bipyramidal coordination geometry.

## Chemical context   

As a natural amino acid, l-histidine occurs in all organisms. It is a metal chelator in plants accumulating nickel from the soil (Krämer *et al.*, 1996 ▸) and a part of the copper-transport system in human blood (Deschamps *et al.*, 2005 ▸). Considerable efforts have been made to combine amino acids with organic and inorganic matrices to produce materials having a non-centrosymmetric cell, large polarizabilities and a strong non-linear optical coefficient (Ben Ahmed *et al.*, 2008 ▸). As a chelating ligand, l-histidine provides up to three potential binding sites, as has been shown in complexes with nickel(II) (Sakurai *et al.*, 1978 ▸), chromium(III) (Pennington *et al.*, 1984 ▸), cobalt(III) (Herak *et al.*, 1981 ▸), molybdenum(V) (Wu *et al.*, 2005 ▸), vanadium(IV) (Islam *et al.*, 2007 ▸) and copper(II) (Deschamps *et al.*, 2005 ▸). In this work, we report the synthesis and structure of the title cadmium complex with l-histidine, (I). Cadmium is structurally inter­esting as it exhibits a number of coordination numbers and geometries such as those in [CdCl_4_] (Boufas *et al.*, 2009 ▸), [Cd_3_Cl_11_] (Kurawa *et al.*, 2008 ▸), [CdCl_6_]_*n*_ (Jarboui *et al.*, 2011 ▸) and [CdCl_4_]_*n*_ (Loseva *et al.*, 2010 ▸).
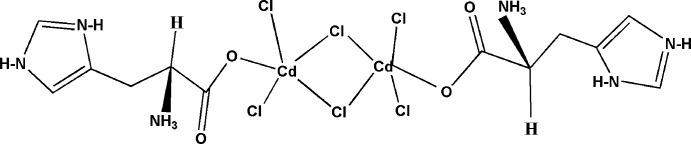


## Structural commentary   

**Figure 1:**
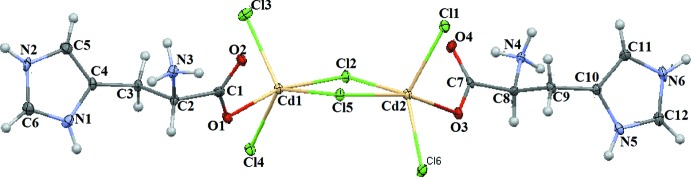
The asymmetric unit of (I), with displacement ellipsoids drawn at the 50% probability level.

The mol­ecular structure of the title compound is shown in Fig. 1 ▸. The asymmetric unit contains a [Cd_2_Cl_6_]^2−^ anionic core bridging a pair of histidinium cations *via* Cd—O bonds. Each cadmium atom is five-coordinated within a CdCl_4_O environment, where atoms Cl3, Cl4 and Cl5 define the equatorial plane for Cd1, and Cl2 and O1 are in axial positions [O1—Cd1—Cl2 = 166.2 (1)°]. A similar coordination is observed for Cd2, and in this case the equatorial plane is formed by atoms Cl1, Cl2 and Cl6, while O3 and Cl5 are in equatorial positions [O3—Cd2—Cl5 = 165.2 (1)°]. Two μ-Cl atoms lead to a Cd_2_Cl_2_ square with a Cd1⋯Cd2 distance of 3.9162 (4) Å. The Cd—Cl distances lie in the range 2.4662 (12) to 2.7244 (14) Å for Cd1 and 2.4812 (11) to 2.7344 (14) Å for Cd2. The Cl—Cd—Cl angles are in the range of 82.61 (4) to 121.93 (3)°.

In the histidinium cation, the α-amino and imidazole groups are protonated and positively charged, while the carboxyl group is deprotonated and negatively charged, which confirms that cations occur in their zwitterionic forms and carry a net positive charge. As expected, the imidazol rings are almost planar with r.m.s deviations for the non-H atoms of 0.003 Å in each mol­ecule. The imidazol group is *trans* to the carboxyl group and *gauche* to the amino N atom.

The conformation of the histidine side chain can be described by the two torsion angles, χ_1_ and χ_21_ (IUPAC–IUB Commission on Biochemical Nomenclature, 1970 ▸). Angle χ_1_, which defines the disposition of the side chain with respect to the main chain, can take values in the neighbourhood of −60, +60 or 180°, corresponding to the open conformation I (*g* ^−^), closed conformation (*g* ^+^) and open conformation II (*t*), respectively (Krause *et al.*, 1991 ▸). The χ_21_ values lie near −90 or +90° but the angle often deviates from these ideal values, as a result of inter­actions between the imidazole ring and other groups in the structure. In the title compound, the following values are seen: χ_1_ = −52.9 (6); χ_1′_ = −52.3 (5); χ_21_ = −72.2 (7); χ_21′_ = −82.5 (7)°. Hence, both histidinium cations adopt the sterically favourable open conformation in (I).

## Supra­molecular features   

**Figure 2:**
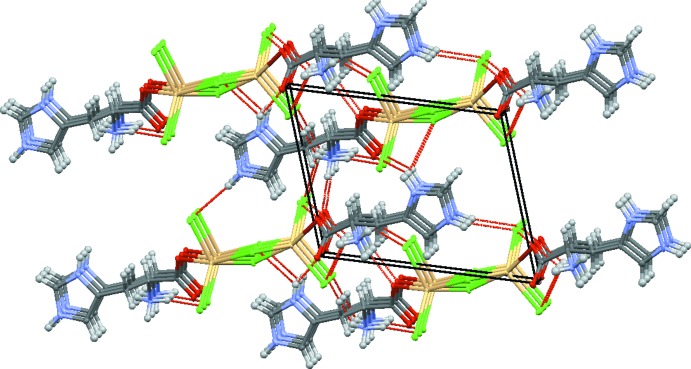
Packing diagram for (I). Red dashed lines indicate hydrogen bonds.

The extended structure of (I) is consolidated by a number of hydrogen-bonding (N—H⋯Cl and N—H⋯O) inter­actions (Table 1 ▸). The chloride anions and oxygen atoms play an important role in accepting hydrogen bonds from the amine N atom and the N atoms of the imidazolium ring. These inter­actions, together with weak C—H⋯Cl and C—H⋯O inter­actions, generate a three-dimensional network (Fig. 2 ▸).

## Database survey   

A search of the Cambridge Structural Database (Version 5.38, update May 2017; Groom *et al.*, 2016 ▸) revealed that the geometric parameters of the title compound are similar to those found in bis(creatininium) tetra­chlorido­cadmate(II) (Boufas *et al.*, 2009 ▸). The imidazol group conformation of the title compound is in contrast to the bent *gauche* conformation found in the structure of l-HisH^+^·Cl^−^·H_2_O (Donohue *et al.*, 1956 ▸, 1964 ▸), but similar to the *trans* conformation observed in dl-HisH^+^·Cl^−^·2H_2_O (Steiner, 1996 ▸)

## Synthesis and crystallization   

The title compound was prepared by dissolving 1 mmol (155.16 mg) of l-histidine in 50.0 ml of water with a mixture of CdCl_2_·2H_2_O (1 mmol) and HCl (8 mmol). The resulting mixture was capped and then heated at 353 K in a water bath for 1 h under continuous stirring and then left to slowly evaporate at room temperature. After two weeks, colourless crystals were obtained, which appear to be indefinitely stable when stored in air. Theoretical calculations and spectroscopic data are available as supporting information.

## Refinement   

Crystal data, data collection and structure refinement details are summarized in Table 2 ▸. All H atoms were located in difference-Fourier maps and subsequently treated as riding atoms in geometrically idealized positions: N—H = 0.86 (NH) or 0.89 (NH_3_) Å, C—H = 0.93 (cyclic), 0.97 (CH_2_) or 0.98 (aliphatic C—H) Å with *U*_iso_(H) = *kU*_eq_(N,C), where *k* = 1.5 for the NH_3_ and methyl groups (which were permitted to rotate but not to tilt) and 1.2 for all other H atoms.

## Supplementary Material

Crystal structure: contains datablock(s) I, _Global. DOI: 10.1107/S205698901900690X/hb7815sup1.cif

Structure factors: contains datablock(s) I. DOI: 10.1107/S205698901900690X/hb7815Isup2.hkl

experimental and theoritical data of the title compound. DOI: 10.1107/S205698901900690X/hb7815sup3.docx

CCDC reference: 1915915

Additional supporting information:  crystallographic information; 3D view; checkCIF report

## Figures and Tables

**Table 1:** Hydrogen-bond geometry (Å, °)

*D*—H⋯*A*	*D*—H	H⋯*A*	*D*⋯*A*	*D*—H⋯*A*
N1—H1*A*⋯O3^i^	0.89	2.01	2.881 (5)	167
N1—H1*C*⋯Cl6^i^	0.89	2.44	3.070 (4)	128
N2—H2⋯Cl4^i^	0.86	2.37	3.215 (5)	167
N3—H3⋯O4^ii^	0.86	1.99	2.751 (6)	146
N4—H11*A*⋯Cl1^iii^	0.89	2.38	3.229 (4)	160
N4—H11*B*⋯Cl3^iv^	0.89	2.70	3.426 (5)	140
N4—H11*C*⋯O1^v^	0.89	1.96	2.846 (6)	171
N5—H22*A*⋯O2^iv^	0.86	1.94	2.699 (6)	147
N6—H33⋯Cl6^v^	0.86	2.28	3.137 (5)	174
C2—H2*A*⋯O4^ii^	0.98	2.56	3.252 (7)	128
C5—H5⋯Cl2^i^	0.93	2.79	3.424 (6)	126
C9—H33*B*⋯Cl3^vi^	0.97	2.81	3.686 (6)	151
C11—H55⋯Cl5^v^	0.93	2.71	3.405 (6)	132
C11—H55⋯O1^v^	0.93	2.53	3.245 (7)	134
C12—H66⋯Cl1^iv^	0.93	2.78	3.618 (6)	151

Symmetry codes: (i) 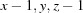; (ii) 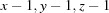; (iii) 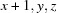; (iv) 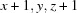; (v) 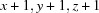; (vi) 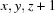.

**Table 2:** Experimental details

Crystal data	
Chemical formula	[Cd_2_(C_6_H_9_N_3_O_2_)_2_Cl_6_]
*M*_r_	749.87
Crystal system, space group	Triclinic, *P*1
Temperature (K)	100
*a*, *b*, *c* (Å)	7.1540 (6), 8.2591 (6), 10.4459 (8)
α, β, γ (°)	108.502 (2), 97.499 (2), 94.512 (2)
*V* (Å^3^)	575.54 (8)
*Z*	1
Radiation type	Mo *K*α
μ (mm^−1^)	2.58
Crystal size (mm)	0.08 × 0.03 × 0.02

Data collection	
Diffractometer	Bruker Nonius KappaCCD
Absorption correction	Multi-scan (*SADABS*; Sheldrick, 2003 ▸)
*T*_min_, *T*_max_	0.820, 0.950
No. of measured, independent and observed [*I* > 2σ(*I*)] reflections	7735, 4856, 4799
*R*_int_	0.025
(sin θ/λ)_max_ (Å^−1^)	0.651

Refinement	
*R*[*F* ^2^ > 2σ(*F* ^2^)], *wR*(*F* ^2^), *S*	0.024, 0.057, 1.08
No. of reflections	4856
No. of parameters	274
No. of restraints	3
H-atom treatment	H-atom parameters constrained
Δρ_max_, Δρ_min_ (e Å^−3^)	1.33, −0.44
Absolute structure	Flack & Bernardinelli (2000 ▸)
Absolute structure parameter	0.02 (2)

Computer programs: *COLLECT*(Nonius, 2002 ▸), *DENZO* and *SCALEPACK* (Otwinowski & Minor, 1997 ▸), *SIR2014* (Burla *et al.*, 2015 ▸), *SHELXL2018* (Sheldrick, 2015 ▸), *Mercury* (Macrae *et al.*, 2006 ▸), *WinGX* (Farrugia, 2012 ▸) and *PARST* (Nardelli, 1995 ▸).

## supplementary crystallographic information

### Crystal data

**Table:**

[Cd_2_(C_6_H_9_N_3_O_2_)_2_Cl_6_]	*Z* = 1
*M**_r_* = 749.87	*F*(000) = 364
Triclinic, *P*1	*D*_x_ = 2.163 Mg m^−^^3^
Hall symbol: P 1	Mo *K*α radiation, λ = 0.71073 Å
*a* = 7.1540 (6) Å	Cell parameters from 6833 reflections
*b* = 8.2591 (6) Å	θ = 2.6–27.6°
*c* = 10.4459 (8) Å	µ = 2.58 mm^−^^1^
α = 108.502 (2)°	*T* = 100 K
β = 97.499 (2)°	Block, colourless
γ = 94.512 (2)°	0.08 × 0.03 × 0.02 mm
*V* = 575.54 (8) Å^3^	

### Data collection

**Table:**

Bruker Nonius KappaCCD diffractometer	4856 independent reflections
Radiation source: fine-focus sealed tube	4799 reflections with *I* > 2σ(*I*)
Graphite monochromator	*R*_int_ = 0.025
Detector resolution: 8.2632 pixels mm^-1^	θ_max_ = 27.6°, θ_min_ = 2.6°
ω scans	*h* = −9→9
Absorption correction: multi-scan (SADABS; Sheldrick, 2003)	*k* = −10→10
*T*_min_ = 0.820, *T*_max_ = 0.950	*l* = −13→13
7735 measured reflections	

### Refinement

**Table:**

Refinement on *F*^2^	Hydrogen site location: inferred from neighbouring sites
Least-squares matrix: full	H-atom parameters constrained
*R*[*F*^2^ > 2σ(*F*^2^)] = 0.024	*w* = 1/[σ^2^(*F*_o_^2^) + (0.0263*P*)^2^] where *P* = (*F*_o_^2^ + 2*F*_c_^2^)/3
*wR*(*F*^2^) = 0.057	(Δ/σ)_max_ < 0.001
*S* = 1.08	Δρ_max_ = 1.33 e Å^−^^3^
4856 reflections	Δρ_min_ = −0.43 e Å^−^^3^
274 parameters	Absolute structure: Flack & Bernardinelli (2000)
3 restraints	Absolute structure parameter: 0.02 (2)

### Special details

**Table:**

Geometry. All esds (except the esd in the dihedral angle between two l.s. planes) are estimated using the full covariance matrix. The cell esds are taken into account individually in the estimation of esds in distances, angles and torsion angles; correlations between esds in cell parameters are only used when they are defined by crystal symmetry. An approximate (isotropic) treatment of cell esds is used for estimating esds involving l.s. planes.

### Fractional atomic coordinates and isotropic or equivalent isotropic displacement parameters (Å^2^)

**Table:**

	*x*	*y*	*z*	*U*_iso_*/*U*_eq_	
Cd2	0.32732 (4)	0.13067 (3)	0.53489 (3)	0.00958 (10)	
Cd1	0.21319 (4)	−0.04006 (3)	0.13444 (3)	0.00944 (10)	
Cl2	0.5118 (2)	0.10427 (15)	0.34159 (13)	0.0111 (3)	
Cl4	0.38809 (19)	−0.29705 (15)	0.05541 (13)	0.0118 (2)	
Cl5	0.0240 (2)	0.02992 (15)	0.32637 (13)	0.0107 (3)	
Cl1	0.25293 (19)	0.42775 (14)	0.63993 (13)	0.0131 (3)	
Cl6	0.18541 (19)	−0.11633 (15)	0.59302 (13)	0.0138 (3)	
Cl3	0.2694 (2)	0.17319 (16)	0.01896 (15)	0.0161 (3)	
O3	0.5872 (5)	0.1400 (4)	0.6964 (4)	0.0117 (7)	
C4	−0.3126 (9)	−0.3001 (7)	−0.4445 (6)	0.0096 (12)	
O4	0.7438 (5)	0.3680 (4)	0.6649 (4)	0.0133 (8)	
O1	−0.0490 (5)	−0.2181 (4)	−0.0211 (4)	0.0115 (7)	
N1	−0.4829 (6)	−0.1360 (5)	−0.1962 (4)	0.0126 (9)	
H1A	−0.447402	−0.043754	−0.218017	0.019*	
H1B	−0.522967	−0.102932	−0.115773	0.019*	
H1C	−0.576801	−0.202840	−0.260150	0.019*	
O2	−0.2172 (6)	0.0068 (4)	0.0144 (4)	0.0133 (8)	
C8	0.8474 (9)	0.3019 (6)	0.8662 (6)	0.0101 (11)	
H22	0.891581	0.194267	0.870762	0.012*	
N5	0.8879 (7)	0.3255 (6)	1.1983 (5)	0.0115 (10)	
H22A	0.860404	0.215366	1.170454	0.014*	
N2	−0.4575 (7)	−0.3273 (6)	−0.6510 (5)	0.0124 (10)	
H2	−0.502332	−0.302327	−0.721887	0.015*	
N3	−0.3875 (7)	−0.4694 (6)	−0.5153 (5)	0.0130 (10)	
H3	−0.378659	−0.553255	−0.483665	0.016*	
N4	1.0146 (6)	0.4309 (5)	0.8826 (4)	0.0107 (9)	
H11A	1.054037	0.413115	0.802492	0.013*	
H11B	1.108038	0.420170	0.943384	0.013*	
H11C	0.981433	0.536510	0.911830	0.013*	
C3	−0.2016 (8)	−0.2381 (6)	−0.3028 (5)	0.0097 (11)	
H3A	−0.138831	−0.122866	−0.284998	0.012*	
H3B	−0.103314	−0.311671	−0.298755	0.012*	
C6	−0.4754 (9)	−0.4837 (7)	−0.6399 (6)	0.0135 (12)	
H6	−0.537559	−0.584164	−0.706536	0.016*	
C9	0.7299 (8)	0.3710 (6)	0.9811 (6)	0.0097 (11)	
H33A	0.669343	0.465858	0.966065	0.012*	
H33B	0.630052	0.280691	0.974320	0.012*	
C1	−0.1857 (10)	−0.1393 (7)	−0.0503 (6)	0.0117 (13)	
C5	−0.3564 (9)	−0.2120 (7)	−0.5316 (6)	0.0147 (13)	
H5	−0.323972	−0.094758	−0.513781	0.018*	
C2	−0.3185 (8)	−0.2340 (6)	−0.1874 (6)	0.0090 (11)	
H2A	−0.362841	−0.351433	−0.192256	0.011*	
C10	0.8396 (9)	0.4311 (7)	1.1223 (6)	0.0095 (12)	
N6	0.9961 (7)	0.5842 (6)	1.3260 (5)	0.0129 (10)	
H33	1.051801	0.670205	1.395165	0.015*	
C12	0.9823 (9)	0.4198 (7)	1.3198 (6)	0.0137 (12)	
H66	1.030715	0.378824	1.388932	0.016*	
C7	0.7185 (9)	0.2673 (7)	0.7288 (6)	0.0086 (12)	
C11	0.9078 (9)	0.5944 (6)	1.2053 (6)	0.0136 (12)	
H55	0.896359	0.694526	1.183723	0.016*	

### Atomic displacement parameters (Å^2^)

**Table:**

	*U*^11^	*U*^22^	*U*^33^	*U*^12^	*U*^13^	*U*^23^
Cd2	0.0101 (2)	0.00984 (15)	0.0073 (2)	−0.00029 (13)	−0.00021 (15)	0.00184 (13)
Cd1	0.0105 (2)	0.00944 (15)	0.0071 (2)	0.00022 (13)	−0.00009 (15)	0.00171 (13)
Cl2	0.0106 (7)	0.0129 (5)	0.0080 (6)	−0.0004 (5)	−0.0004 (5)	0.0020 (5)
Cl4	0.0112 (7)	0.0124 (5)	0.0097 (6)	0.0026 (5)	0.0001 (5)	0.0013 (4)
Cl5	0.0106 (7)	0.0127 (5)	0.0071 (6)	0.0004 (5)	0.0007 (5)	0.0016 (5)
Cl1	0.0155 (7)	0.0120 (5)	0.0111 (6)	0.0024 (5)	0.0040 (6)	0.0021 (5)
Cl6	0.0123 (7)	0.0171 (5)	0.0111 (6)	−0.0051 (5)	−0.0026 (5)	0.0069 (5)
Cl3	0.0145 (7)	0.0195 (5)	0.0185 (7)	0.0018 (5)	0.0022 (6)	0.0123 (5)
O3	0.012 (2)	0.0110 (14)	0.0090 (18)	−0.0013 (13)	−0.0035 (15)	0.0016 (13)
C4	0.008 (3)	0.013 (2)	0.007 (3)	0.001 (2)	0.002 (2)	0.001 (2)
O4	0.015 (2)	0.0168 (16)	0.0083 (19)	0.0010 (15)	−0.0007 (16)	0.0057 (14)
O1	0.012 (2)	0.0102 (14)	0.0091 (18)	0.0019 (14)	−0.0026 (15)	0.0000 (13)
N1	0.012 (2)	0.0147 (18)	0.011 (2)	0.0012 (17)	0.0015 (19)	0.0044 (17)
O2	0.016 (2)	0.0105 (14)	0.0098 (19)	0.0018 (13)	−0.0032 (16)	0.0005 (13)
C8	0.012 (3)	0.009 (2)	0.009 (3)	−0.003 (2)	−0.002 (2)	0.004 (2)
N5	0.011 (3)	0.0110 (18)	0.010 (3)	0.0024 (17)	0.003 (2)	0.0005 (17)
N2	0.014 (3)	0.019 (2)	0.006 (2)	0.0066 (19)	0.004 (2)	0.0040 (19)
N3	0.011 (3)	0.017 (2)	0.012 (2)	0.0009 (17)	0.001 (2)	0.0072 (18)
N4	0.014 (2)	0.0109 (16)	0.007 (2)	0.0009 (16)	0.0019 (18)	0.0030 (15)
C3	0.010 (3)	0.012 (2)	0.006 (3)	0.0005 (19)	0.001 (2)	0.0022 (19)
C6	0.010 (3)	0.016 (2)	0.012 (3)	−0.002 (2)	0.001 (2)	0.003 (2)
C9	0.009 (3)	0.009 (2)	0.010 (3)	0.0004 (18)	0.003 (2)	0.0012 (19)
C1	0.013 (3)	0.010 (2)	0.012 (3)	−0.001 (2)	0.001 (2)	0.005 (2)
C5	0.017 (3)	0.014 (2)	0.013 (3)	0.004 (2)	0.002 (3)	0.004 (2)
C2	0.009 (3)	0.012 (2)	0.006 (3)	0.003 (2)	0.002 (2)	0.002 (2)
C10	0.009 (3)	0.010 (2)	0.009 (3)	0.001 (2)	0.003 (2)	0.003 (2)
N6	0.012 (3)	0.015 (2)	0.008 (3)	−0.0009 (19)	0.001 (2)	−0.0014 (19)
C12	0.014 (3)	0.016 (3)	0.009 (3)	0.002 (2)	0.001 (2)	0.002 (2)
C7	0.010 (3)	0.008 (2)	0.006 (3)	0.001 (2)	0.002 (2)	−0.001 (2)
C11	0.017 (3)	0.012 (2)	0.012 (3)	0.000 (2)	0.003 (2)	0.004 (2)

### Geometric parameters (Å, º)

**Table:**

Cd1—O1	2.361 (4)	N5—C10	1.388 (6)
Cd1—Cl3	2.4662 (12)	N5—H22A	0.8600
Cd1—Cl5	2.5061 (14)	N2—C6	1.331 (7)
Cd1—Cl4	2.5155 (12)	N2—C5	1.374 (8)
Cd1—Cl2	2.7244 (14)	N2—H2	0.8600
Cd2—O3	2.320 (3)	N3—C6	1.335 (7)
Cd2—Cl1	2.4812 (11)	N3—H3	0.8600
Cd2—Cl6	2.4864 (12)	N4—H11A	0.8900
Cd2—Cl2	2.5155 (14)	N4—H11B	0.8900
Cd2—Cl5	2.7344 (14)	N4—H11C	0.8900
Cd2—Cd1	3.9162 (5)	C3—C2	1.548 (8)
O3—C7	1.280 (7)	C3—H3A	0.9700
C4—C5	1.355 (7)	C3—H3B	0.9700
C4—N3	1.383 (7)	C6—H6	0.9300
C4—C3	1.494 (8)	C9—C10	1.486 (8)
O4—C7	1.236 (6)	C9—H33A	0.9700
O1—C1	1.274 (7)	C9—H33B	0.9700
N1—C2	1.489 (6)	C1—C2	1.543 (9)
N1—H1A	0.8900	C5—H5	0.9300
N1—H1B	0.8900	C2—H2A	0.9800
N1—H1C	0.8900	C10—C11	1.362 (8)
O2—C1	1.236 (7)	N6—C12	1.334 (7)
C8—N4	1.492 (7)	N6—C11	1.366 (7)
C8—C7	1.531 (8)	N6—H33	0.8600
C8—C9	1.542 (8)	C12—H66	0.9300
C8—H22	0.9800	C11—H55	0.9300
N5—C12	1.319 (8)		
			
O1—Cd1—Cl3	99.31 (8)	C8—N4—H11A	109.5
O1—Cd1—Cl5	91.97 (9)	C8—N4—H11B	109.5
Cl3—Cd1—Cl5	118.96 (4)	H11A—N4—H11B	109.5
O1—Cd1—Cl4	84.88 (8)	C8—N4—H11C	109.5
Cl3—Cd1—Cl4	113.33 (4)	H11A—N4—H11C	109.5
Cl5—Cd1—Cl4	127.41 (4)	H11B—N4—H11C	109.5
O1—Cd1—Cl2	166.16 (8)	C4—C3—C2	115.5 (5)
Cl3—Cd1—Cl2	94.41 (4)	C4—C3—H3A	108.4
Cl5—Cd1—Cl2	82.99 (4)	C2—C3—H3A	108.4
Cl4—Cd1—Cl2	87.97 (4)	C4—C3—H3B	108.4
O3—Cd2—Cl1	97.93 (9)	C2—C3—H3B	108.4
O3—Cd2—Cl6	85.65 (9)	H3A—C3—H3B	107.5
Cl1—Cd2—Cl6	121.93 (4)	N2—C6—N3	107.2 (5)
O3—Cd2—Cl2	95.70 (10)	N2—C6—H6	126.4
Cl1—Cd2—Cl2	112.59 (4)	N3—C6—H6	126.4
Cl6—Cd2—Cl2	124.74 (4)	C10—C9—C8	115.2 (5)
O3—Cd2—Cl5	165.13 (8)	C10—C9—H33A	108.5
Cl1—Cd2—Cl5	96.33 (4)	C8—C9—H33A	108.5
Cl6—Cd2—Cl5	83.27 (4)	C10—C9—H33B	108.5
Cl2—Cd2—Cl5	82.61 (4)	C8—C9—H33B	108.5
Cd2—Cl2—Cd1	96.64 (5)	H33A—C9—H33B	107.5
Cd1—Cl5—Cd2	96.62 (5)	O2—C1—O1	127.5 (6)
C7—O3—Cd2	117.6 (3)	O2—C1—C2	116.9 (5)
C5—C4—N3	105.7 (5)	O1—C1—C2	115.5 (5)
C5—C4—C3	129.8 (5)	C4—C5—N2	107.6 (5)
N3—C4—C3	124.5 (4)	C4—C5—H5	126.2
C1—O1—Cd1	114.9 (3)	N2—C5—H5	126.2
C2—N1—H1A	109.5	N1—C2—C1	108.2 (4)
C2—N1—H1B	109.5	N1—C2—C3	110.9 (4)
H1A—N1—H1B	109.5	C1—C2—C3	107.0 (5)
C2—N1—H1C	109.5	N1—C2—H2A	110.2
H1A—N1—H1C	109.5	C1—C2—H2A	110.2
H1B—N1—H1C	109.5	C3—C2—H2A	110.2
N4—C8—C7	110.6 (4)	C11—C10—N5	105.7 (5)
N4—C8—C9	109.8 (4)	C11—C10—C9	129.2 (4)
C7—C8—C9	108.1 (5)	N5—C10—C9	125.1 (5)
N4—C8—H22	109.4	C12—N6—C11	109.2 (5)
C7—C8—H22	109.4	C12—N6—H33	125.4
C9—C8—H22	109.4	C11—N6—H33	125.4
C12—N5—C10	109.6 (5)	N5—C12—N6	108.0 (5)
C12—N5—H22A	125.2	N5—C12—H66	126.0
C10—N5—H22A	125.2	N6—C12—H66	126.0
C6—N2—C5	109.5 (5)	O4—C7—O3	126.8 (6)
C6—N2—H2	125.3	O4—C7—C8	118.0 (5)
C5—N2—H2	125.3	O3—C7—C8	115.0 (4)
C6—N3—C4	110.1 (4)	C10—C11—N6	107.4 (4)
C6—N3—H3	125.0	C10—C11—H55	126.3
C4—N3—H3	125.0	N6—C11—H55	126.3
			
C5—C4—N3—C6	−0.7 (7)	C4—C3—C2—N1	−52.9 (6)
C3—C4—N3—C6	−178.8 (5)	C4—C3—C2—C1	−170.8 (4)
C5—C4—C3—C2	110.2 (7)	C12—N5—C10—C11	−0.8 (7)
N3—C4—C3—C2	−72.2 (7)	C12—N5—C10—C9	−179.0 (6)
C5—N2—C6—N3	0.1 (7)	C8—C9—C10—C11	99.8 (7)
C4—N3—C6—N2	0.4 (7)	C8—C9—C10—N5	−82.5 (7)
N4—C8—C9—C10	−52.3 (5)	C10—N5—C12—N6	0.5 (7)
C7—C8—C9—C10	−173.0 (4)	C11—N6—C12—N5	0.1 (7)
Cd1—O1—C1—O2	−18.4 (8)	Cd2—O3—C7—O4	−16.1 (8)
Cd1—O1—C1—C2	157.2 (3)	Cd2—O3—C7—C8	159.6 (4)
N3—C4—C5—N2	0.7 (7)	N4—C8—C7—O4	−13.9 (7)
C3—C4—C5—N2	178.7 (6)	C9—C8—C7—O4	106.4 (6)
C6—N2—C5—C4	−0.5 (7)	N4—C8—C7—O3	169.9 (4)
O2—C1—C2—N1	−11.4 (7)	C9—C8—C7—O3	−69.8 (5)
O1—C1—C2—N1	172.5 (5)	N5—C10—C11—N6	0.9 (7)
O2—C1—C2—C3	108.2 (6)	C9—C10—C11—N6	178.9 (6)
O1—C1—C2—C3	−67.8 (6)	C12—N6—C11—C10	−0.6 (7)

### Hydrogen-bond geometry (Å, º)

**Table:**

*D*—H···*A*	*D*—H	H···*A*	*D*···*A*	*D*—H···*A*
N1—H1*A*···O3^i^	0.89	2.01	2.881 (5)	167
N1—H1*C*···Cl6^i^	0.89	2.44	3.070 (4)	128
N2—H2···Cl4^i^	0.86	2.37	3.215 (5)	167
N3—H3···O4^ii^	0.86	1.99	2.751 (6)	146
N4—H11*A*···Cl1^iii^	0.89	2.38	3.229 (4)	160
N4—H11*B*···Cl3^iv^	0.89	2.70	3.426 (5)	140
N4—H11*C*···O1^v^	0.89	1.96	2.846 (6)	171
N5—H22*A*···O2^iv^	0.86	1.94	2.699 (6)	147
N6—H33···Cl6^v^	0.86	2.28	3.137 (5)	174
C2—H2*A*···O4^ii^	0.98	2.56	3.252 (7)	128
C5—H5···Cl2^i^	0.93	2.79	3.424 (6)	126
C9—H33*B*···Cl3^vi^	0.97	2.81	3.686 (6)	151
C11—H55···Cl5^v^	0.93	2.71	3.405 (6)	132
C11—H55···O1^v^	0.93	2.53	3.245 (7)	134
C12—H66···Cl1^iv^	0.93	2.78	3.618 (6)	151

Symmetry codes: (i) *x*−1, *y*, *z*−1; (ii) *x*−1, *y*−1, *z*−1; (iii) *x*+1, *y*, *z*; (iv) *x*+1, *y*, *z*+1; (v) *x*+1, *y*+1, *z*+1; (vi) *x*, *y*, *z*+1.
