# Supplementary material for: Crystal structure of di-μ-chlorido-bis­[di­chlorido(l-histidinium-κO)cadmium(II)]
Source: Acta Crystallogr E Crystallogr Commun. 2019 May 17;75(Pt 6):823–5. doi: 10.1107/S205698901900690X (PMC6658965; doi:10.1107/S205698901900690X)
Supplement: Supplementary file 3 [file e-75-00823-sup3.docx]

**Supplementary materials**

**1- Preliminary characterization**

All reagents were purchased commercially and were used without further purification. X-ray diffraction data were collected on a Nonius KappaCCD diffractometer operating with Mo Kα radiation (λ = 0.71073 Å) at a scanning rate of 0.02° s^-1^ from 2.62 to 27.56°. FT-IR spectra (4000-500 cm^-1^) were recorded on a Nicolet iS10 FT-IR spectrophotometer. The absorbance measurements were carried out on a powdered sample of pure (I). The UV diffuse reflectance spectra were measured on a Varian Cary 5000. The Raman spectrum of the single crystal was obtained in the micro-single mode of the T64000 HORIBA Jobin Yvon Raman spectrometer operating with grating set at 1800 grooves/mm. The green line (λ = 633nm) of the argon ion laser served as an excitation source. Spectra were recorded in 10 repetitions with an accumulation time of 60 s.

**2- Computational details**

The density functional (DFT/B3LYP/LanL2DZ) (Lee *et al.*, 1988, Becke, 1993 and Parr *et al.*, 1989) set level was adopted to calculate the properties of the title molecule in this work using Gaussian 09w program package (Frish, *et al.*, 2009). Starting from the X-ray experimental data and in order to take into account the effect of intermolecular interactions, we have considered the cluster built up from one Cd_2_Cl_6_ dimer and two protoned histidine cations. Singlet ground state geometry optimization of this complex was performed with the default convergence criteria without any constraint on the geometry (Schlegel, 1982).

Selected experimental and calculated structural parameters, of both organic and inorganic parts are illustrated in Table 3. As can be shown, the average relative error between observed and calculated bonds length and angle are 1.64% and 2.69 % for the histidinium cation and 5.02 % and 3.65 % for the Cd_2_Cl_6_ dimer, respectively. In their majority, the computed bond lengths are slightly longer than the experimental one. These discrepancies can be explained by the fact that the calculations assume an isolated molecule where the intermolecular Coulombic interaction with the neighboring molecules are absent, whereas the experimental result corresponds to interacting molecules in the crystal lattice. With Gabedit program (Allouche, 2011), we can easily find the features of the highest occupied molecular orbital (HOMO) and the lowest unoccupied molecular orbital (LUMO) of (1). Obviously, the electron –population of the singulet state of the HOMO dominantly resides in the chloride atoms, while that of the LUMO basically locates at the imidazol group.

The band-gap energy value calculated was found to be 4.83 eV (Fig. S1).


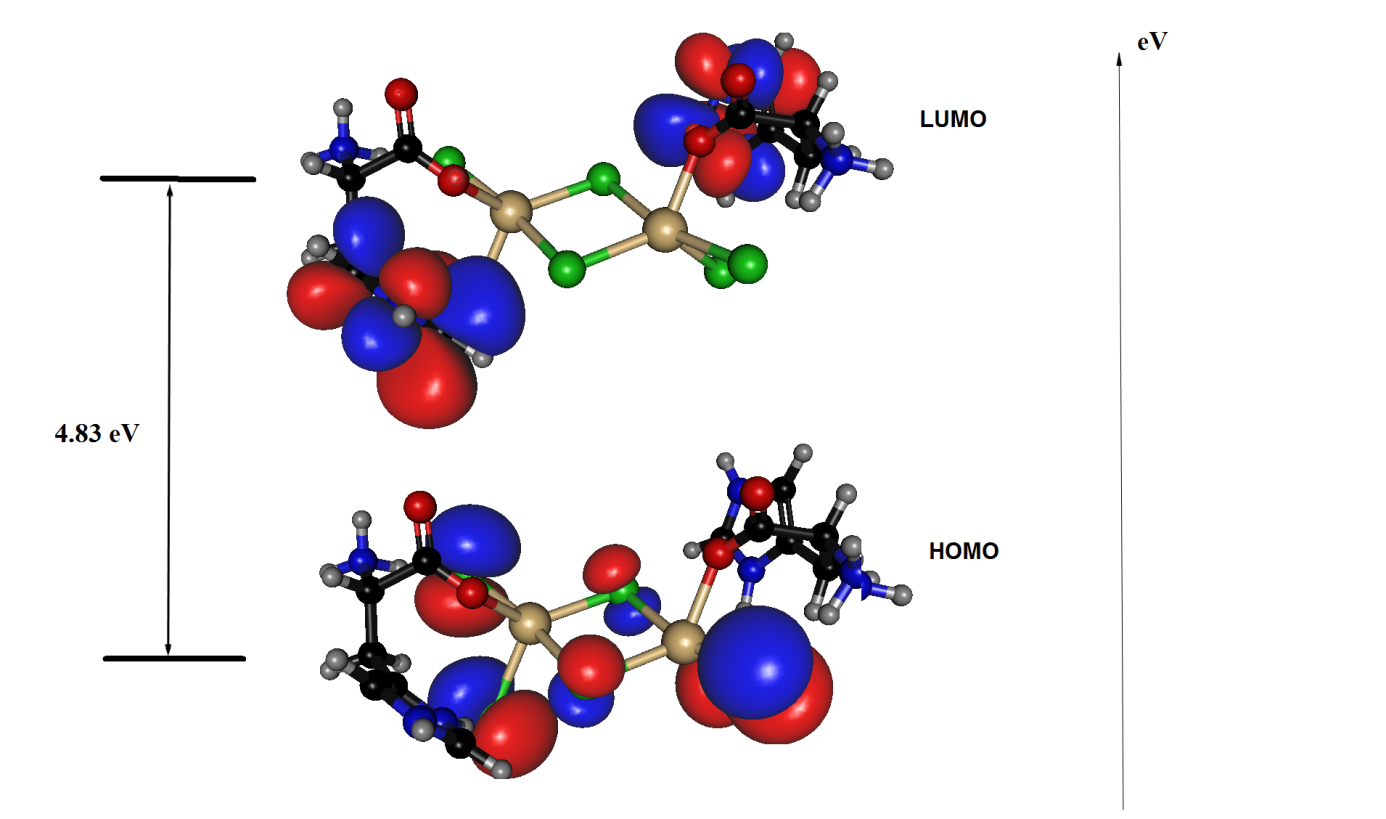


[**Figure S**](readonly)1: The frontier molecular orbitals for the title compound

**3- FT -IR and Raman spectra**

In FT-IR pectrum, the vibration of the title compound appears in the 4000-400 cm^-1^ frequency range. As it can be seen, a noticeable difference between experimental and the calculated can be observed (Fig. S2). frequencies associated to the N-H, C-H, C=O and C-N stretching were found.

The amino acids exist as zwitterions in solution and in solid state. The IR spectra of amino acids exhibited significant features in νNH_3_ and νCOO^-^ regions. The broad band observed at 3137.98 cm^-1^, calculated at 3052 cm^-1^ is assigned to the asymmetric -NH_3_^+^ stretching. The bands observed at 1574.64 and 1493.96 cm^-1^ are assigned respectively to the asymmetric and symmetric stretching modes of COO^-^. The corresponding calculated values are 1608 and 1572 cm^-1^, respectively.

N-H and C-H vibrations are observed in the IR spectrum at 1334.54 cm^-1^ and 1409.24 cm^-1^, calculated at 1396 and 1299 cm^-1^. While, the peak due to imidazole in plane was observed at 826.03 cm^-1^. NH_3_^+^ twisting and rocking and COO^-^ wagging frequencies were observed in the range 1200–600 cm^-1^.

In the case of the external vibrations of the chlorocadmate(II) anion, the asymmetric and symmetric stretching of Cd-Cl appeared at 234 and 92.62 cm ^-1^, respectively. The DFT calculations yielded those modes at 276 and 249 cm ^-1^. The band observed at 205 cm^-1^ was assigned to the Cd-Cl bending mode. The bands corresponding to the deformation mode of Cl-Cd-Cl appeared in the 106–136 m^-1^ region. While the 172 cm^-1^ Raman band was attributed to the Cd-O bending mode. The lattice modes were observed in the Raman spectra at 70 cm ^-1^ (Fig. S3).

It is worth noting that in our case there is an additional potential source of differences between the experimental and calculated spectra other than the usual systematic ones, resulting from the approximate nature of level of theory used, and the large extent compensated by the use of scaling factors. In fact, due to these reasons, our calculations were made by analyzing only one cluster built up from two C_6_N_3_O_2_H_9_ cations and one Cd_2_Cl_6_ anion, disregarding possible lattice effects.

The infrared and Raman study confirms the presence of the organic group C_6_N_3_O_2_H_9_^+^ and the tetrahedral anion Cd_2_Cl_6_^2-^.


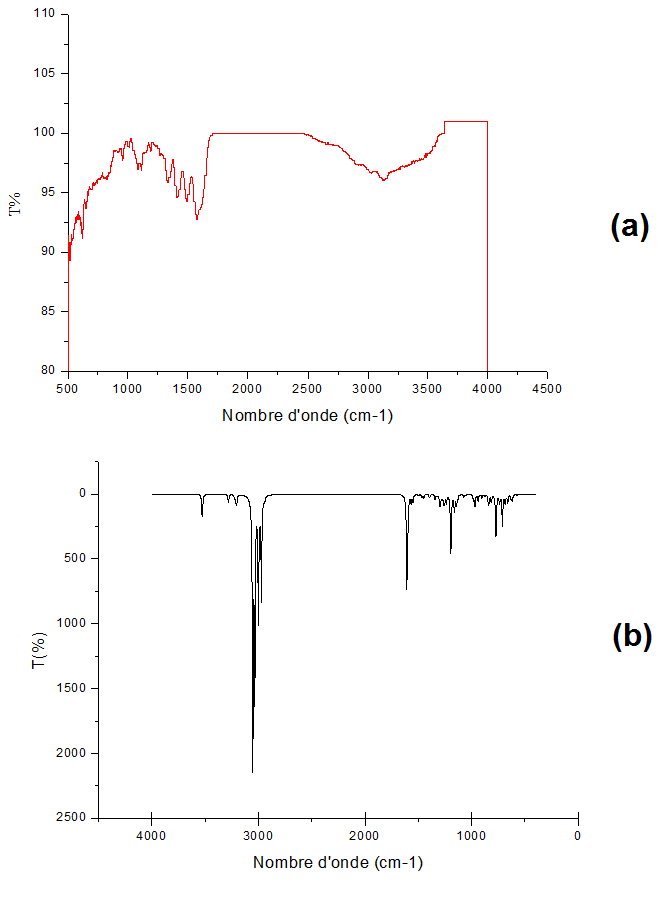


[**Figure S**](readonly)2: (a) The experimental FT-IR spectra and (b) simulated FT-IR spectra of the title compound at room temperature between 4000 and 400 cm^-1^ .


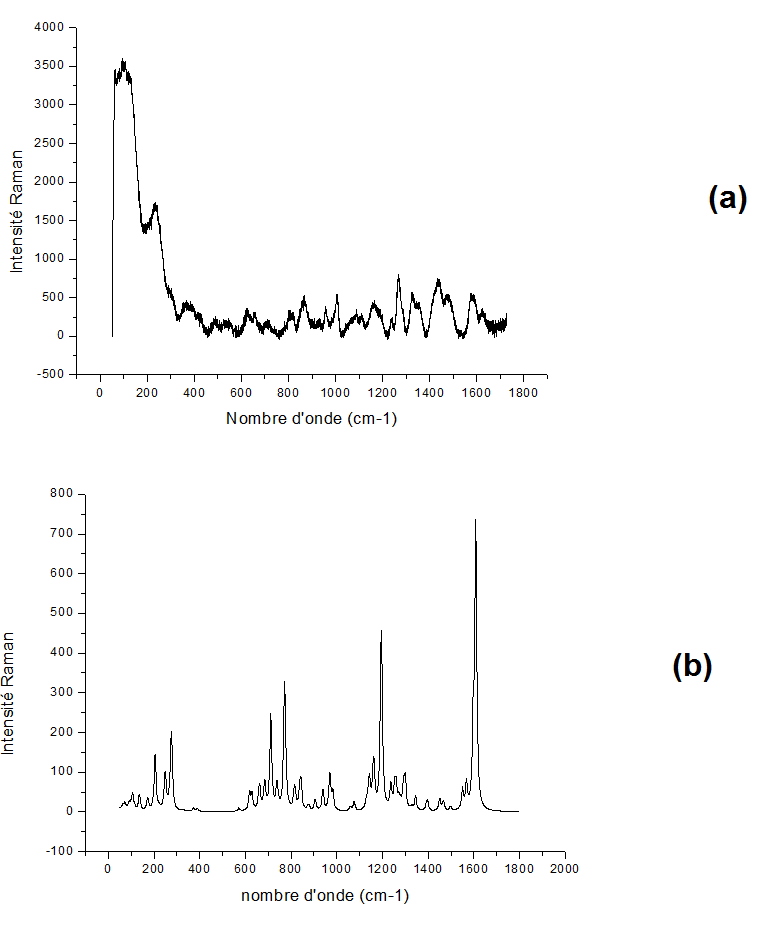


[**Figure S3:**](readonly) (a) The experimental Raman spectra and (b) simulated Raman spectra of the title compound measured at room temperature in the region 50 – 1800 cm^-1^.

**4- UV absorption**

Scientists have reported that a common and convenient method for determining whether a band gap is indirect or direct is to use the UV–Vis absorption spectrum (Guo *et al.*, 2011).

The absorption spectrum obtained in the visible region is shown in Fig. S4 (see Supporting information).in the title complex, UV spectrum exhibits one strong shoulder at λmax = 210.1 nm, which is smoothly displaced with respect to the L-histidine band, λmax = 209.6 nm. The broad absorption band observed for the compound may be due to the n π* and π π* transitions for the imidazole ring. However, in the theoretical spectrum, λmax = 230 nm. This difference is due to the reasons cited in FT -IR and Raman spectra section.


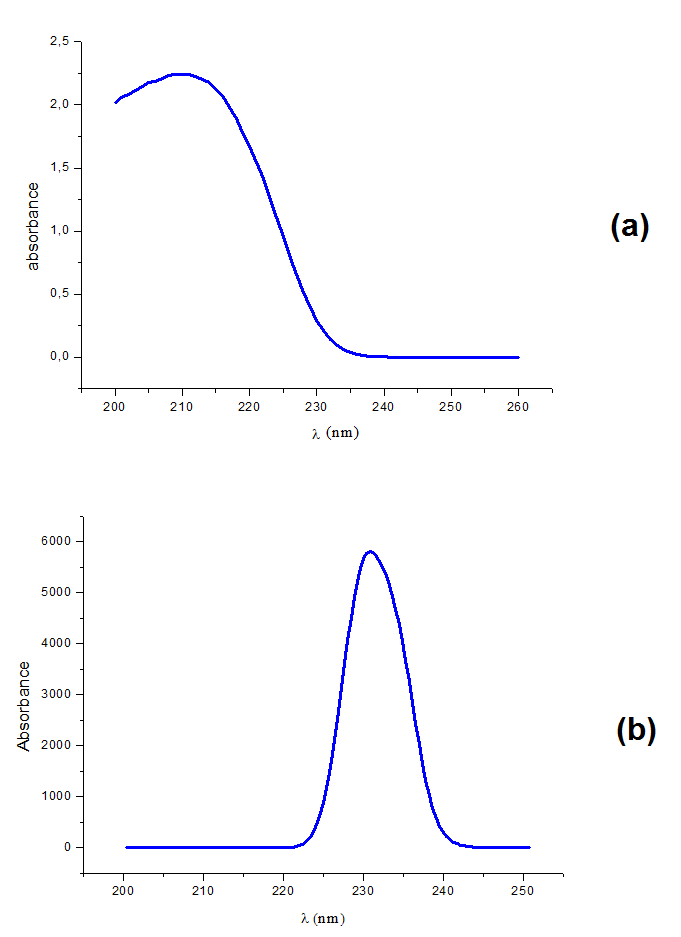


**Figure S4:** (a) The experimental UV spectra and (b) simulated UV spectra of the title compound measured at room temperature in the region 200 – 250 cm^-1^.
